# The relationships between Life’s Crucial 9, Life’s Essential 8, and sarcopenia in older adults: multicenter case–control study

**DOI:** 10.3389/fmed.2025.1714385

**Published:** 2025-12-01

**Authors:** Hui Li, Huaqing Liu, Jialin Zhang, Hui Nie, Shunnan Wang, Zhibang Zhao, Gengze Wang

**Affiliations:** 1 Department of Gastroenterology, Nanyang Central Hospital, Nanyang, Henan, China; 2 Department of Nursing, Nanyang Central Hospital, Nanyang, Henan, China; 3 Emergency Trauma Center, Nanyang Second People’s Hospital, Nanyang, Henan, China

**Keywords:** Life’s Crucial 9, Life’s Essential 8, sarcopenia, muscle mass, grip strength, mental health, PHQ-9, cardiovascular health

## Abstract

**Background:**

Sarcopenia, the age-related loss of muscle mass and strength, is aggravated by cardiometabolic and psychosocial burden. Life’s Essential 8 (LE8) and its mental-health extension Life’s Crucial 9 (LC9) quantify cardio-psycho-metabolic risk, yet their relationship with sarcopenia remains untested.

**Methods:**

Multicenter case–control study of 1,316 consecutive inpatients (≥65 years) enrolled 2019–2025 at two tertiary hospitals in Nanyang, China (Nanyang Central Hospital, *n* = 740; Nanyang Second People’s Hospital, *n* = 576). Sarcopenia was adjudicated by AWGS-2019. LE8 and LC9 were derived from admission nicotine, diet, sleep, physical activity, BMI, blood pressure, glucose, non-HDL-C, and PHQ-9. Multivariable logistic regression, ROC, and re-classification analyses were performed, adjusted for age, sex, BMI, hemoglobin, albumin, smoking, and comorbidity.

**Results:**

Overall sarcopenia prevalence 11.81–14.05%. Mean LC9 was lower in sarcopenic versus non-sarcopenic participants. ROC curves showed LC9 discriminated sarcopenia better than LE8. Across low, moderate, and high categories, sarcopenia prevalence descended steeply with LC9 and modestly with LE8. Multivariable analysis confirmed both scores as independent protectors.

**Conclusion:**

LC9, a freely calculable metric integrating mental health with cardio-metabolic health, is strongly and independently associated with sarcopenia in hospitalized older adults and outperforms LE8. Routine LC9 assessment at admission may enable early identification and multimodal intervention to prevent muscle loss.

## Introduction

Sarcopenia, originally defined as the age-related loss of skeletal muscle mass, is now recognized as a multidimensional geriatric syndrome characterized by progressive and generalized decline in muscle strength, quantity, and function (1). The revised 2019 consensus of the European Working Group on Sarcopenia in Older People (EWGSOP2) emphasizes low muscle strength as the principal diagnostic parameter, with confirmation by reduced muscle quantity or quality, and identifies poor physical performance as an indicator of disease severity (2). Beyond its direct contribution to frailty, falls, and loss of autonomy, sarcopenia is associated with increased cardiometabolic morbidity, hospitalization, and all-cause mortality (3). Despite its clinical importance, preventive strategies remain limited, partly because modifiable determinants that operate across the life-course are still incompletely understood (4).

Recently, the American Heart Association (AHA) introduced Life’s Essential 8 (LE8) as an updated metric of cardiovascular health (CVH). LE8 comprises four health behaviors (diet, physical activity, nicotine exposure, and sleep duration) and four cardiometabolic factors (body-mass index, non-HDL cholesterol, blood glucose, and blood pressure), each scored 0–100 and aggregated into a composite 0–100 scale (5). Compared with its predecessor, Life’s Simple 7, LE8 incorporates sleep health and adopts more stringent, evidence-based thresholds, thereby capturing a broader spectrum of lifestyle-related risk (6). Life’s Crucial 9 (LC9) is an extended construct of cardiovascular health (CVH) that builds upon the American Heart Association’s Life’s Essential 8 (LE8) by incorporating mental health as a ninth component (7). The integration of psychological health into CVH metrics reflects growing recognition of mental well-being as a critical determinant of cardiovascular and systemic health outcomes (8).

Both Life’s Essential 8 (LE8) and Life’s Crucial 9 (LC9) are designed as quantifiable, modifiable, and population-level metrics primarily aimed at optimizing cardiovascular health. However, their relevance likely extends beyond cardiovascular protection (9, 10). Emerging evidence suggests that several individual components of LE8 and LC9—particularly physical activity, dietary quality, glycemic control, and adiposity—intersect with key molecular pathways involved in muscle homeostasis (11, 12). These include the regulation of muscle protein turnover, mitochondrial biogenesis, and systemic inflammatory responses (13–15). Dysregulation in these pathways is recognized as a central mechanism in the pathogenesis of sarcopenia (16, 17). Therefore, it is plausible that higher cardiovascular health scores, as reflected by LE8 or LC9, may be associated with better muscle function and mass, such as greater grip strength and appendicular lean mass, in middle-aged and older adults. Collectively, these observations suggest a potential link between LE8/LC9 and the risk or progression of sarcopenia. Nevertheless, empirical studies directly examining this relationship remain scarce, particularly in non-Western populations.

To address this knowledge gap, we conducted a multicenter, population-based cohort study across clinical centers, enrolling a large sample of older adults aged ≥65 years. By leveraging high-quality longitudinal data and standardized assessments of both LE8 and LC9 metrics and sarcopenia-related outcomes, our study aims to evaluate the relationship between cumulative cardiovascular health—reflected by LE8 and LC9 scores—and the risk of prevalent sarcopenia. We hypothesize that sustained adherence to optimal CVH profiles, including mental well-being, is independently associated with a reduced risk of sarcopenia development. The findings from this study may provide robust evidence supporting the integration of cardiovascular and mental health promotion into strategies for sarcopenia prevention and healthy aging.

## Methods

### Study design and population

This multicenter, case–control study was conducted between May 2019 and May 2025 at two tertiary hospitals in Nanyang City, Henan Province, China: the Department of Gastroenterology, Nanyang Central Hospital (NCH), and the Emergency Trauma Center, Nanyang Second People’s Hospital (NSPH). Consecutive patients aged ≥65 years admitted to either center were screened for eligibility. The study was approved by the Institutional Ethics Committee of both participating hospitals (NCH: ID: 20190771 and NSPH: 2020 Research Review No. 12), and written informed consent was obtained from all participants.

Inclusion criteria: (i) age ≥65 years on the date of admission; (ii) willingness to provide written informed consent; and (iii) availability of complete anthropometric and grip-strength data required for sarcopenia classification. Exclusion criteria: (i) presence of active malignant; (ii) severe cognitive impairment precluding reliable interview; (iii) unavailable measurements of HGS and ASM.

**Figure 1:**
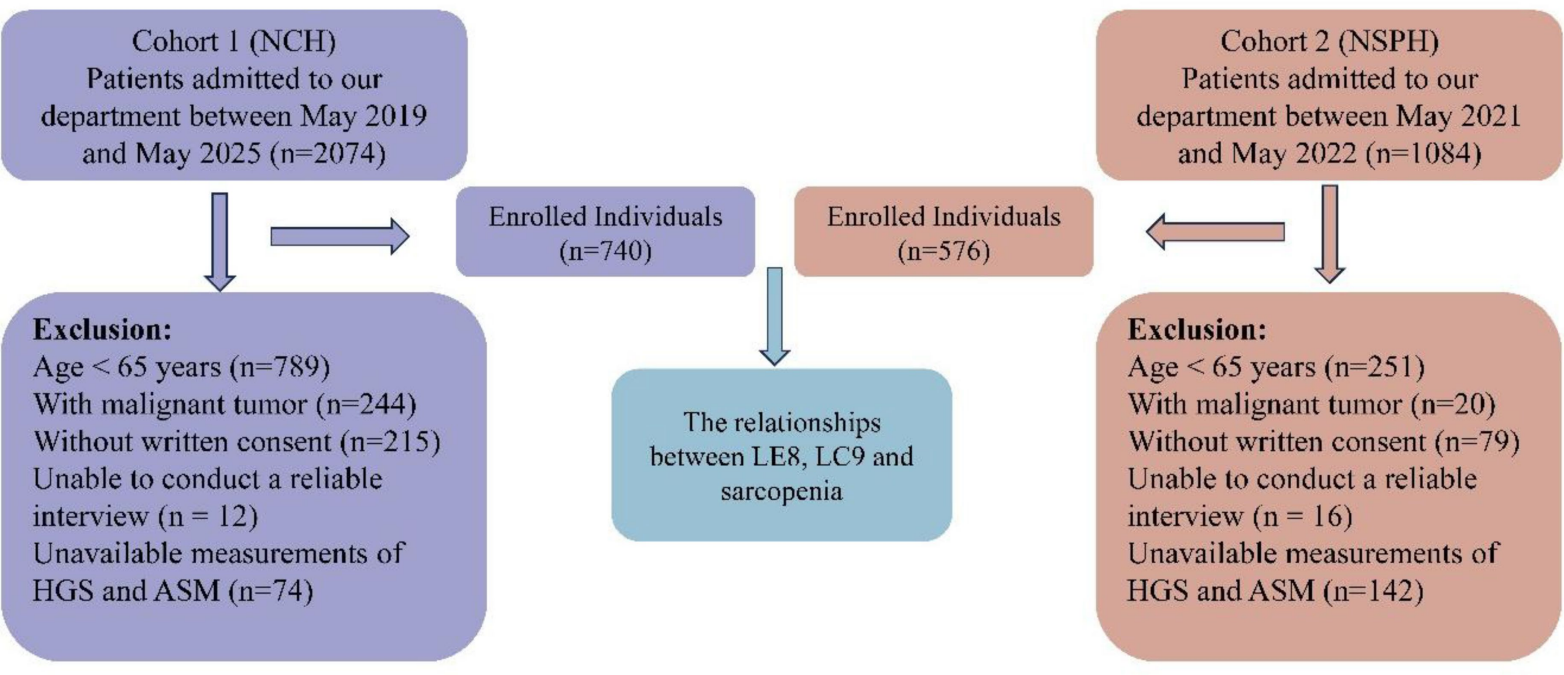
Flow chart of our study.

During the recruitment window, 2,074 patients were screened at NCH and 1,084 at NSPH. After applying the above criteria, participants with any missing value among the eight LE8 components, PHQ-9, grip strength, ASM or covariates were excluded, and 740 and 576 eligible individuals were enrolled from NCH and NSPH, respectively, yielding a final analytic cohort of 1,316 older adults (Figure 1).

### Clinical data and baseline measurements

Within 24 h of admission, trained research personnel used a standardized electronic case-report form to collect all baseline variables. Age was recorded in completed years. BMI was computed as weight (kg) divided by height squared (m^2^). Sex, smoking history, and alcoholism history were obtained by a structured interview. The Charlson Comorbidity Index (CCI) was calculated from discharge diagnoses; a score >4 was classified as high comorbidity burden (18). A 12-lead resting electrocardiogram and a postero-anterior chest radiograph were performed on the index day; abnormalities were adjudicated by senior radiologists and cardiologists blinded to sarcopenia status. Hypertension was defined as prior physician diagnosis, current use of antihypertensive drugs, or supine blood pressure ≥140/90 mmHg measured twice after 5 min rest. Fasting venous blood was drawn on the first morning after admission. Red-blood-cell count (RBC, ×10^12^ L^−1^), hemoglobin (Hb, g L^−1^), albumin (ALB, g L^−1^), and fasting plasma glucose (GLU, mmol L^−1^) were measured. All assays were completed within 2 h of sampling.

### Definitions and measurement of LE8 and LC9

We operationalized Life’s Essential 8 (LE8) and Life’s Crucial 9 (LC9) according to the 2022 American Heart Association (AHA) presidential advisory and its 2024 psychosocial extension, respectively, with minor adaptations for a Chinese inpatient population (19, 20). The metrics were calculated from variables collected within 24 h of admission, plus fasting blood samples drawn on the morning after admission. LE8 sums eight equally-weighted items (0–100 each): four behaviors (nicotine, diet, sleep, physical activity) and four biological factors (BMI, blood pressure, fasting glucose, non-HDL-C), and PHQ-9 was used for evaluating the mental health of LC9. PHQ-9 total scores were categorized as 100 (0–4, none-minimal), 75 (5–9, mild), 50 (10–14, moderate), 25 (15–19, moderately severe), and 0 (20–27, severe) following previous studies (21). The nine domain scores were averaged to generate an overall LE8 or LC9 score (0–100). Following AHA recommendations, participants were classified as having high (80–100), moderate (50–79), or low (0–49) cardiovascular health.

### Sarcopenia assessment

Sarcopenia was adjudicated according to the 2019 Asian Working Group for Sarcopenia (AWGS) algorithm (2). Hand grip strength (HGS) was quantified with a spring-loaded dynamometer; the highest reading (kg) was retained; low strength was defined as <28 kg (men) or <18 kg (women). Appendicular skeletal muscle mass (ASM) was estimated by eight-point, multi-frequency bio-impedance (InBody BWA2.0, Shanghai) at 1 kHz–3 MHz, which has been validated against DXA in Chinese community-dwelling elderly (22). Electrodes were positioned at the wrist and ankle bilaterally; impedance was recorded in all four limbs and summed by the manufacturer’s equation. ASM was computed as ASM (kg)/height^2^ (m^2^); cut-offs for low muscle mass were <7.0 kg m^−2^ (men) and <5.7 kg m^−2^ (women). Sarcopenia required both low HGS and low ASM.

### Statistical analysis

Continuous variables are expressed as mean ± standard deviation (SD); normality was formally tested with the Shapiro–Wilk test. Categorical variables are summarized as frequencies and proportions. Between-group comparisons for continuous variables were performed with the two-sample Student’s *t* test (parametric) or the Mann–Whitney *U* test (non-parametric), as appropriate. Proportions were compared with the χ^2^ test, or Fisher’s exact test when any expected cell count was <5. LE8 and LC9 scores were investigated both as continuous (per 5-point increment) and as categorical variables (low 0–49, moderate 50–79, high 80–100) based on AHA cut-offs. Receiver-operating-characteristic (ROC) curves were constructed for LE8 and LC9 to predict sarcopenia risk; the area under the ROC curve (AUC) with 95% confidence interval (CI) is reported. Crude and multivariable-adjusted logistic regression models were fitted to estimate odds ratios (ORs) and 95% CIs for the cross-sectional association of LE8 or LC9 with sarcopenia. Linear regression was employed to quantify the association of LE8/LC9 with continuous muscle outcomes (HGS and ASM). To circumvent collinearity, LE8 and LC9 were evaluated in separate regression models; we report two distinct sets of analyses rather than simultaneous inclusion of both scores. All statistical tests were two-tailed; *p* < 0.05 was considered statistically significant. Analyses were performed with R version 4.3.2 (R Foundation for Statistical Computing, Vienna, Austria).

## Results

### Baseline characteristics

As illustrated in Figure 1, 3,158 consecutive inpatients aged ≥65 years were screened (2,074 at Nanyang Central Hospital [NCH] between May 2019 and May 2025; 1,084 at Nanyang Second People’s Hospital [NSPH] between May 2021 and May 2022). After application of uniform exclusion criteria, 740 and 576 participants were finally enrolled from NCH and NSPH, respectively, yielding an analytical cohort of 1,316 older adults.

Supplementary Tables S1, S2 summarize the baseline profile of each center. Across both cohorts, sarcopenic participants were older, had lower grip strength and appendicular skeletal muscle mass, and exhibited consistently poorer cardiovascular health scores. In the NCH cohort (*n* = 740; 104 sarcopenic), the patients with sarcopenia were older than normal individuals (78.4 ± 8.5 vs. 76.3 ± 8.8 years, *p* = 0.010), while more females and smokers were identified in the normal group. Moreover, in sarcopenia group, Hb and ALB were lower (80.8 ± 22 vs. 88.1 ± 25 g L^−1^, *p* = 0.007; 33.5 ± 12.5 vs. 36.6 ± 13.5 g L^−1^, *p* = 0.020), and both LE8 and LC9 scores were markedly reduced (LE8: 63.3 ± 12.3 vs. 67.6 ± 10.3, *p* = 0.001; LC9: 60.4 ± 11.6 vs. 68.4 ± 10.8, *p* < 0.001).

Comparable differences were observed in NSPH (*n* = 576; 68 sarcopenic). Sarcopenic individuals were older still (85.2 ± 8.7 vs. 76.7 ± 8.9 years, *p* < 0.001), had higher BMI, and again displayed significantly lower LE8 (61.4 ± 13.1 vs. 66.6 ± 10.4, *p* = 0.001) and LC9 (58.3 ± 13.0 vs. 67.4 ± 10.1, *p* < 0.001) scores. Other variables did not differ consistently between sarcopenic and non-sarcopenic subjects.

### Comparison of LE8 and LC9 scores across muscle status groups

**Figure 2:**
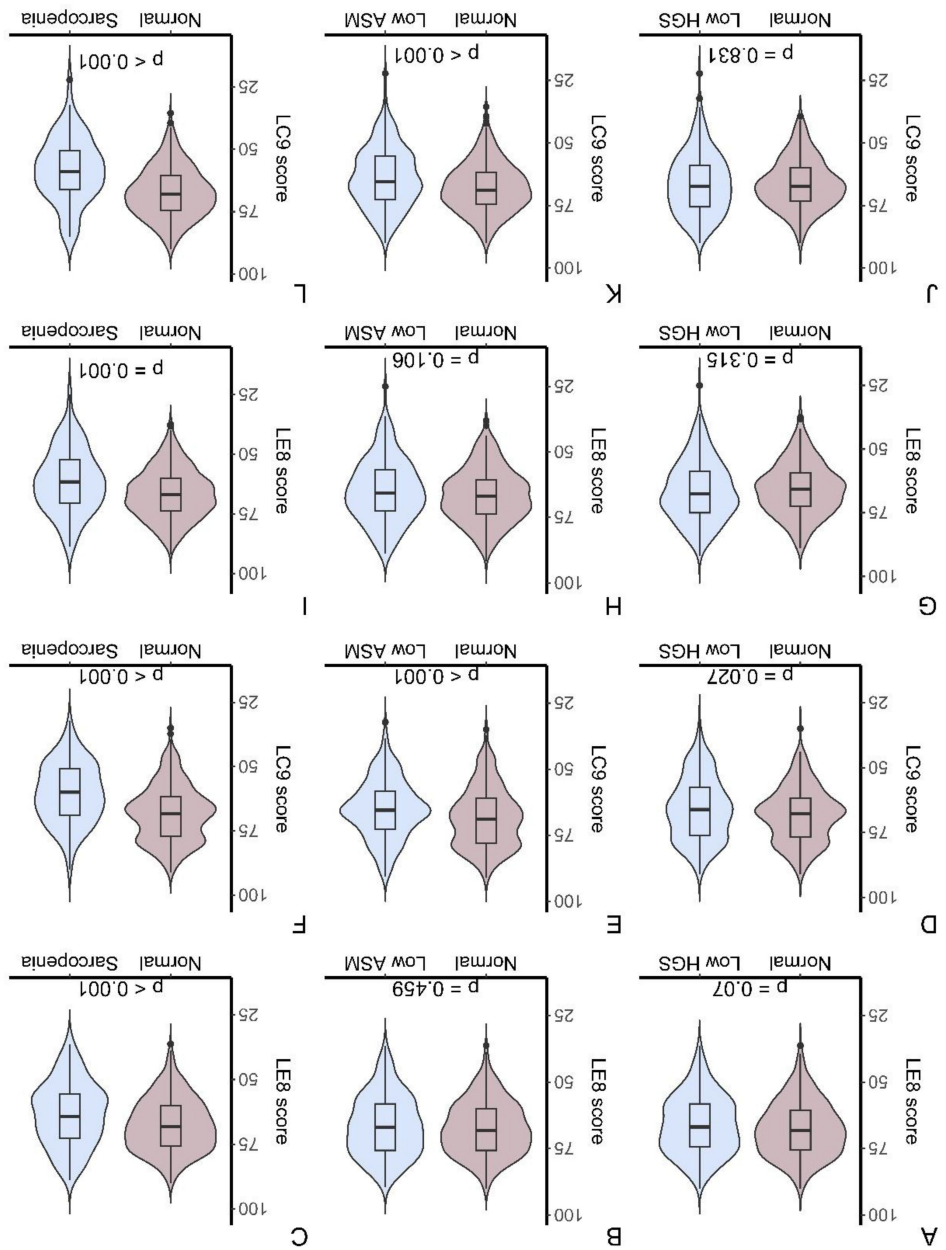
LE8 and LC9 scores across muscle status categories in older patients. **(A–C)** LE8 scores in NCH cohort among participants with low HGS only, low ASM only, or sarcopenia; **(D–F)** LC9 scores in NCH cohort among participants with low HGS only, low ASM only, or sarcopenia; **(G–I)** LE8 scores in NSPH cohort among participants with low HGS only, low ASM only, or sarcopenia; **(J–L)** LC9 scores in NSPH cohort among participants with low HGS only, low ASM only, or sarcopenia.

Figure 2 depicts LE8 and LC9 total scores across three mutually exclusive muscle status categories: low handgrip strength (HGS) only, low appendicular skeletal muscle mass (ASM) only, and sarcopenia, separately for each center. In the NCH cohort (Figures 2A–F), Participants with sarcopenia had the lower LE8 score, while LC9 scores differed significantly across all three groups (low HGS vs. normal, *p* = 0.027; low ASM vs. normal, *p* < 0.001; sarcopenia vs. normal, *p* < 0.001). Findings were replicated in the NSPH cohort (Figures 2G–L). The patients with sarcopenia had significantly lower LE8 scores and L9 scores (LE8, *p* = 0.001; LC9, *p* < 0.001).

### ROC curves of LE8 and LC9 for sarcopenia

**Figure 3:**
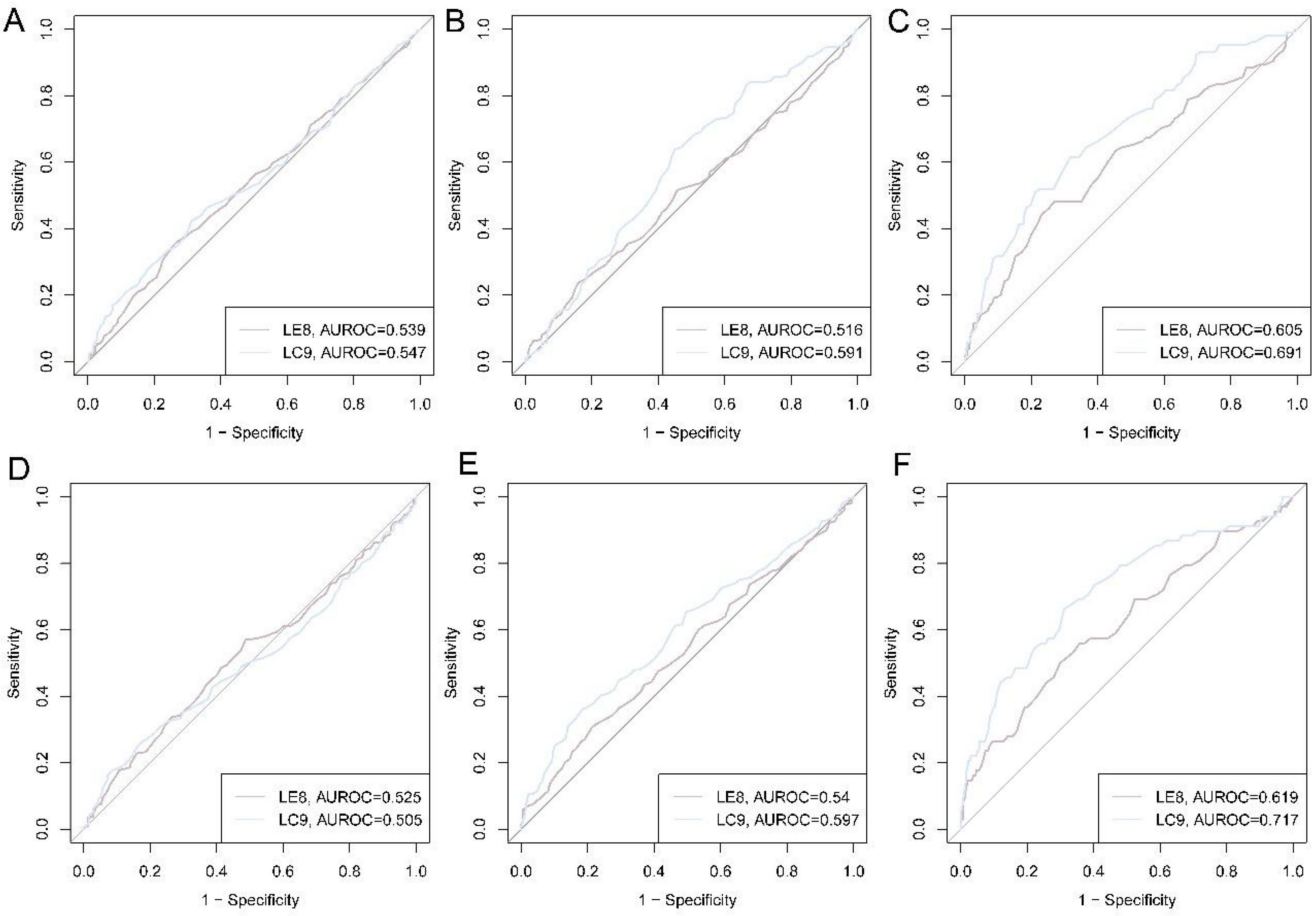
ROC curves of LE8 and LC9 for low HGS, low ASM, and sarcopenia. **(A)** ROC curves for low HGS in NCH cohort; **(B)** ROC curves for low ASM in NCH cohort; **(C)** ROC curves for sarcopenia in NCH cohort; **(D)** ROC curves for low HGS in NSPH cohort; **(E)** ROC curves for low ASM in NSPH cohort; **(F)** ROC curves for sarcopenia in NSPH cohort.

The receiver-operating-characteristic (ROC) curves for LE8 and LC9 in relation to three muscle-related traits (HGS, ASM, and sarcopenia) are shown in Figure 3. In NCH (Figures 3A–C), the discriminative capacity of LE8 was modest for low HGS (AUC 0.539) and low ASM (AUC 0.516), but improved noticeably for the composite sarcopenia outcome (AUC 0.605). LC9 performed consistently better: AUCs were 0.547 for low HGS, 0.591 for low ASM, and 0.691 for sarcopenia. Findings were replicated in NSPH (Figures 3D–F). LE8 AUCs were 0.525 for low HGS, 0.540 for low ASM, and 0.619 for sarcopenia. Corresponding LC9 AUCs were 0.505, 0.597, and 0.717. LC9 may have a higher AUC in sarcopenia than LE8.

### Sarcopenia traits of patients with different LE8 and LC9 scores

Following AHA recommendations, LE8 and LC9 were also operationalized as categorical variables (low, middle, and high), and the sarcopenia traits of patients with different LE8 and LC9 scores were summarized in Table 1. In the NCH cohort, sarcopenia prevalence across LE8 categories (low, moderate, high) was 32.6, 12.8 and 13.4% (*p* = 0.001); the corresponding figures for LC9 were 37.7, 13.3 and 4.7% (*p* < 0.001). A similar gradient was observed in the NSPH cohort: LE8 28.9, 10.6, 8.5% (*p* = 0.001); LC9 38.1, 9.7, 10.2% (*p* < 0.001).

**Table 1:** HGS, ASM, and sarcopenia of patients with different LE8 and LC9 scores.

NCH cohort									
Outcomes	Overall	LE8 (low)	LE8 (middle)	LE8 (high)	*p*	LC9 (low)	LC9 (middle)	LC9 (high)	*p*
	(*n* = 740)	(*n* = 46)	(*n* = 612)	(*n* = 82)		(*n* = 61)	(*n* = 573)	(*n* = 106)	
HGS (kg)	24.73 ± 11.86	21.54 ± 10.26	25.03 ± 11.96	24.26 ± 11.81	0.147	20.23 ± 9.45	25.05 ± 11.97	25.55 ± 12.12	0.008
ASM (kg/m2)	7.08 ± 2.60	6.43 ± 2.17	7.16 ± 2.62	6.79 ± 2.63	0.103	6.48 ± 2.30	7.00 ± 2.62	7.83 ± 2.54	0.002
Low HGS	319 (43.11%)	25 (54.35%)	260 (42.48%)	34 (41.46%)	0.278	40 (65.57%)	237 (41.36%)	42 (39.62%)	0.001
Low ASM	285 (38.51%)	23 (50.00%)	226 (36.93%)	36 (43.90%)	0.121	29 (47.54%)	227 (39.62%)	29 (27.36%)	0.019
Sarcopenia	104 (14.05%)	15 (32.61%)	78 (12.75%)	11 (13.41%)	0.001	23 (37.70%)	76 (13.26%)	5 (4.72%)	<0.001

NSPH cohort									
Outcomes	Overall	LE8 (low)	LE8 (middle)	LE8 (high)	*p*	LC9 (low)	LC9 (middle)	LC9 (high)	*p*
	(*n* = 576)	(*n* = 45)	(*n* = 472)	(*n* = 59)		(*n* = 42)	(*n* = 475)	(*n* = 59)	
HGS (kg)	24.47 ± 11.76	21.89 ± 11.44	25.01 ± 11.76	22.03 ± 11.66	0.057	20.76 ± 10.68	25.19 ± 11.75	21.29 ± 11.71	0.006
ASM (kg/m2)	7.00 ± 2.61	6.33 ± 2.45	7.09 ± 2.63	6.77 ± 2.58	0.134	5.75 ± 2.30	7.10 ± 2.63	7.06 ± 2.50	0.006
Low HGS	237 (41.15%)	20 (44.44%)	185 (39.19%)	32 (54.24%)	0.077	20 (47.62%)	182 (38.32%)	35 (59.32%)	0.006
Low ASM	219 (38.02%)	22 (48.89%)	173 (36.65%)	24 (40.68%)	0.246	25 (59.52%)	173 (36.42%)	21 (35.59%)	0.012
Sarcopenia	68 (11.81%)	13 (28.89%)	50 (10.59%)	5 (8.47%)	0.001	16 (38.10%)	46 (9.68%)	6 (10.17%)	<0.001

ASM, appendicular skeletal muscle mass index; HGS, hand-grip strength; LE8, Life’s Essential 8; LC9, Life’s Crucial 9; NCH, Nanyang Central Hospital; NSPH, Nanyang Second People’s Hospital.

Continuous muscle outcomes mirrored these findings. Mean ASM in NCH increased linearly across LC9 tertiles (6.48 vs. 7.00 vs. 7.83 kg m^−2^, *p* = 0.002); HGS improved from 20.2 to 25.6 kg (*p* = 0.008). Comparable trends were seen in NSPH (ASM: 5.75 vs. 7.10 vs. 7.06 kg m^−2^, *p* = 0.006; HGS: 20.8 vs. 25.2 vs. 21.3 kg, *p* = 0.006). However, no significant differences in HGS and ASM were estimated in individuals grouped by LE8 (all *p* > 0.05). Collectively, the data demonstrate that LC9 provides superior discrimination and clinical stratification for sarcopenia than LE8 alone, with a robust dose–response across the full spectrum of cardiovascular, metabolic, and mental health.

### Multivariable associations of LE8 and LC9 with sarcopenia traits

To reduce the bias caused by co-variables, multivariable models were established. Table 2 summarizes linear regression estimates for continuous HGS and ASM, while Supplementary Table S3 and Table 3 present univariable and multivariable logistic models for low HGS, low ASM, and sarcopenia. The multivariate models were adjusted for the variables that were significant in baseline characteristics. For NCH cohorts, age, sex, Hb, smoking, and ALB were included, and for NSPH cohorts, the models were adjusted for age, sex, and BMI. After full adjustment, both continuous LE8 and LC9 remained positively associated with both HGS and ASM in NCH, while the associations were not significant in NSPH (Table 3). In NCH, compared with high LC9, low LC9 is associated with reduced HGS and ASM; low LE8 showed no independent effect. Similarly, in NSPH, low LC9 was associated with lower ASM, whereas LE8 categories were not significant.

**Table 2:** Linear regression of LE8 and LC9 scores for HGS and ASM.

NCH cohort								
Variables	HGS				ASM			
	Univariate		Multivariate		Univariate		Multivariate	
	β	*p*	β	*p*	β	*p*	β	*p*
LE8 (continuous)	0.097	0.018	0.116	0.01	0.016	0.078	0.021	0.04
LC9 (continuous)	0.119	0.002	0.135	0.001	0.039	<0.001	0.047	<0.001
LE8 (low)	−2.713	0.214	−2.881	0.207	−0.361	0.451	−0.465	0.355
LE8 (middle)	0.773	0.579	0.764	0.585	0.375	0.22	0.344	0.265
LE8 (high)	Ref	Ref	Ref	Ref	Ref	Ref	Ref	Ref
LC9 (low)	−5.318	0.005	−5.666	0.004	−1.349	0.001	−1.545	<0.001
LC9 (middle)	−0.493	0.693	−0.32	0.8	−0.83	0.002	−0.857	0.002
LC9 (high)	Ref	Ref	Ref	Ref	Ref	Ref	Ref	Ref

NSPH cohort								
Variables	HGS				ASM			
	Univariate		Multivariate		Univariate		Multivariate	
	β	*p*	β	*p*	β	*p*	β	*p*
LE8 (continuous)	0.037	0.408	0.016	0.717	0.013	0.201	−0.005	0.789
LC9 (continuous)	0.091	0.043	0.058	0.201	0.042	<0.001	0.003	0.845
LE8 (low)	−0.145	0.95	0.468	0.839	−0.436	0.399	−0.483	0.034
LE8 (middle)	2.981	0.066	2.615	0.102	0.326	0.365	−0.005	0.77
LE8 (high)	Ref	Ref	Ref	Ref	Ref	Ref	Ref	Ref
LC9 (low)	−0.526	0.823	0.849	0.718	−1.306	0.013	−0.446	0.05
LC9 (middle)	3.899	0.016	3.982	0.013	0.044	0.903	−0.005	0.774
LC9 (high)	Ref	Ref	Ref	Ref	Ref	Ref	Ref	Ref

ASM, appendicular skeletal muscle mass index; HGS, hand-grip strength; LE8, Life’s Essential 8; LC9, Life’s Crucial 9; NCH, Nanyang Central Hospital; NSPH, Nanyang Second People’s Hospital.

**Table 3:** Multivariate logistics models of LE8 and LC9 scores for low HGS, low ASM, and sarcopenia.

Variables	NCH cohort					
	Low HGS		Low ASM		Sarcopenia	
	OR [95 %CI]	*p*	OR [95 %CI]	*p*	OR [95 %CI]	*p*
LE8 (continuous)	0.978 [0.962, 0.993]	0.006	0.985 [0.969, 1.000]	0.055	0.942 [0.921, 0.963]	<0.001
LC9 (continuous)	0.976 [0.961, 0.990]	0.001	0.964 [0.949, 0.978]	<0.001	0.916 [0.895, 0.936]	<0.001
LE8 (low)	1.978 [0.902, 4.385]	0.09	1.572 [0.722, 3.438]	0.254	6.697 [2.483, 18.824]	<0.001
LE8 (middle)	1.114 [0.687, 1.823]	0.663	0.805 [0.500, 1.305]	0.374	1.126 [0.581, 2.366]	0.738
LE8 (high)	Ref	Ref	Ref	Ref	Ref	Ref
LC9 (low)	3.587 [1.775, 7.421]	<0.001	3.104 [1.539, 6.331]	0.002	26.057 [9.106, 88.252]	<0.001
LC9 (middle)	1.205 [0.776, 1.886]	0.41	1.975 [1.244, 3.210]	0.005	3.717 [1.591, 10.880]	0.006
LC9 (high)	Ref	Ref	Ref	Ref	Ref	Ref

Variables	NSPH cohort					
	Low HGS		Low ASM		Sarcopenia	
	OR [95 %CI]	*p*	OR [95 %CI]	*p*	OR [95 %CI]	*p*
LE8 (continuous)	1.006 [0.990, 1.022]	0.466	0.986 [0.970, 1.002]	0.09	0.964 [0.940, 0.988]	0.003
LC9 (continuous)	0.999 [0.983, 1.015]	0.918	0.967 [0.951, 0.983]	<0.001	0.932 [0.906, 0.956]	<0.001
LE8 (low)	0.626 [0.279, 1.391]	0.252	1.261 [0.563, 2.833]	0.573	3.678 [1.142, 13.357]	0.035
LE8 (middle)	0.516 [0.294, 0.901]	0.02	0.843 [0.481, 1.497]	0.553	1.425 [0.564, 4.393]	0.491
LE8 (high)	Ref	Ref	Ref	Ref	Ref	Ref
LC9 (low)	0.618 [0.269, 1.404]	0.252	2.544 [1.107, 5.984]	0.029	4.595 [1.517, 15.415]	0.009
LC9 (middle)	0.409 [0.230, 0.718]	0.002	1.036 [0.585, 1.877]	0.905	0.928 [0.381, 2.633]	0.877
LC9 (high)	Ref	Ref	Ref	Ref	Ref	Ref

ASM, appendicular skeletal muscle mass index; HGS, hand-grip strength; LE8, Life’s Essential 8; LC9, Life’s Crucial 9; OR, odds ratio; CI, confidence interval; NCH, Nanyang Central Hospital; NSPH, Nanyang Second People’s Hospital.

Multivariable logistic regression (Table 3) revealed that both continuous LC9 conferred the strongest protection against sarcopenia in both centers (NCH: OR = 0.916; NSPH: OR = 0.932, both *p* < 0.001). LE8 was also significant but with lesser magnitude (NCH: OR = 0.942; NSPH: OR = 0.964, both *p* < 0.05). When analyzed ordinally, low LC9 was associated with a higher risk of sarcopenia in both the NCH and NSPH cohorts relative to high LC9. Low LE8 showed a weaker, center-dependent association (NCH: OR = 6.70, *p* < 0.001; NSPH: OR = 3.68, *p* = 0.035). Comparable associations were also observed for isolated low ASM and low HGS (Table 3). In general, high LE8, as well as high LC9, were estimated as protective factors for sarcopenia and sarcopenia traits even after being adjusted for co-variables.

## Discussion

The present multicenter case–control study contrasted the two most recently proposed composite metrics of cardiovascular health—Life’s Essential 8 (LE8) and its psychosocially expanded derivative Life’s Crucial 9 (LC9)—with respect to sarcopenia risk in hospitalized older adults. Three principal observations emerge. First, both LE8 and LC9 displayed significant, stepwise, inverse associations with sarcopenia, but the magnitude of protection was consistently larger for LC9. Second, the added value of LC9 was not simply statistical; the mental-health domain captured by PHQ-9 meaningfully improved discriminative accuracy (AUC 0.69–0.72 vs. 0.61–0.62 for LE8) and re-classified 25–30% of participants into more appropriate risk strata. Third, the benefits of higher LC9 were independent of age, sex, hemoglobin, albumin, smoking, and BMI, suggesting that a broadly defined “cardio-psycho-metabolic” profile is a modifiable life-course determinant of muscle ageing.

Our findings extend earlier population-based reports that focused on individual components of cardiovascular health. Physical activity, protein-rich diets, glycemic control, and leanness are already linked to larger mid-life muscle mass and slower decline thereafter (23). By integrating these behaviors into a single graded score, we provide clinicians with a ready-to-use metric that correlates with objective muscle outcomes in the very old and multi-morbid. The observation that LC9 outperforms LE8 corroborates the emerging concept that mental well-being operates beyond mood modulation. Depression and chronic stress activate the hypothalamic–pituitary–adrenal axis, raise catabolic cortisol, suppress sex steroids and growth hormone, down-regulate mammalian target of rapamycin complex 1 (mTORC1) signaling, and amplify systemic inflammation—all established mechanisms in sarcopenia pathophysiology (24–26). Quantifying this psychoneuroendocrine burden through a simple 9-item questionnaire and merging it with traditional cardio-metabolic variables, therefore, appears biologically plausible and clinically efficient.

Several mechanistic pathways may link higher LC9 to preserved myocyte integrity. Higher diet quality scores usually reflect greater intakes of n-3 polyunsaturated fatty acids and antioxidant vitamins, which dampen nuclear factor-κB–mediated proteolysis (27). Optimal physical activity stimulates myofibrillar protein synthesis via insulin-like growth factor-1 and mechanistic signaling, while also enhancing mitochondrial oxidative capacity (28, 29). Non-HDL cholesterol and fasting glucose at target ranges limit advanced glycation end-product accumulation and collagen cross-linking within the endomysium, thereby maintaining muscle quality (30, 31). Non-smoking and normotension reduce oxidative stress and microvascular rarefaction, respectively (32). Finally, better sleep duration and mental health mitigate circadian disruption and glucocorticoid excess, both of which accelerate myonuclear apoptosis (23). LC9, by simultaneously favoring these pathways, may create a synergistic milieu that counteracts the “anabolic resistance” typical of ageing muscle.

Strengths of our study include the use of the 2019 AWGS algorithm with direct grip dynamometry and multi-frequency bio-impedance, the harmonization of LE8/LC9 definitions with 2022–2024 AHA guidance, the inclusion of two independent hospital cohorts, and the rigorous adjustment for nutritional (albumin, hemoglobin) and disease-burden indices. Limitations merit acknowledgement. The case–control design precludes causal inference; reverse causation (sub-clinical sarcopenia leading to lower physical activity, poorer diet, and depressive symptoms) is possible. Second, muscle mass was estimated rather than directly quantified by magnetic resonance imaging or dual-energy X-ray absorptiometry. Yet eight-point bio-impedance correlates strongly with DXA-derived appendicular lean mass in Asian older adults, and identical cut-offs are endorsed by AWGS. Third, dietary and sleep data were collected through interviewer-administered questionnaires, introducing recall bias. Future deployment of brief food-frequency or actigraphy validation in a sub-sample could calibrate measurement error. Although lifestyle components of LE8/LC9 are stable behaviors, admission BP, glucose and lipids may transiently deviate from usual values because of acute illness; such short-term fluctuations would most likely attenuate rather than inflate the reported associations. Moreover, participants were recruited from gastroenterology and trauma/emergency wards, where acute inflammation, immobilization and protein loss may transiently amplify muscle deficits. This setting likely overestimates sarcopenia prevalence and could restrict the external validity of our findings to less acute or elective geriatric inpatients. Additionally, the present study operationalized the mental-health component of LC9 exclusively through PHQ-9, a depression-screening instrument that does not evaluate anxiety, stress, resilience or positive well-being. Future investigations should therefore adopt multidomain. Finally, all participants were Han Chinese recruited from two tertiary hospitals in Nanyang, Henan province. The regional characteristics may limit direct extrapolation to Western settings and should be considered when implementing LC9-based risk thresholds.

Clinical implications are twofold. On the screening front, LC9 offers a pragmatic, cost-free tool to flag high-risk individuals who may benefit from a comprehensive sarcopenia work-up. Embedding PHQ-9 into routine admission clerking already satisfies Joint Commission patient-safety goals; coupling its output with seven routine cardio-metabolic variables instantly generates an LC9 score that stratifies sarcopenia probability with 70% accuracy—comparable to frailty indices requiring >30 items. On the interventional front, our data justify testing multi-modal “LC9 optimization bundles” (smoking cessation, Mediterranean-style hospital menus, in-bed cycle ergometry, sleep-hygiene bundles, and stepped depression care) as pre-habilitation before elective procedures or rehabilitation after acute illness. Such trials should employ muscle protein fractional synthesis rates or grip-strength trajectory as mechanistic end-points, and formally compare LC9-targeted therapy with usual nutrition/exercise prescriptions.

In conclusion, LC9—a metric originally conceived for cardiovascular surveillance—robustly captures sarcopenia risk beyond the sum of its parts. The incorporation of mental health into CVH frameworks is not merely academic; it tangibly enhances discriminative power and reveals a modifiable target that clinicians can address with evidence-based psycho-pharmacological or behavioral interventions. If longitudinal studies validate these cross-sectional observations, LC9 could evolve into a life-course indicator that unifies primary prevention of cardiometabolic disease, mood disorders, and skeletal-muscle decline, thereby promoting intrinsic capacity and healthy ageing across populations.

## Data Availability

The raw data supporting the conclusions of this article will be made available by the authors, without undue reservation.
